# Plasma extracellular vesicle neurofilament light chain as the biomarkers of the progression of Parkinson’s disease

**DOI:** 10.17305/bb.2024.11502

**Published:** 2024-12-03

**Authors:** Chien-Tai Hong, Chen-Chih Chung, Yi-Chen Hsieh, Lung Chan

**Affiliations:** 1 Department of Neurology, Shuang Ho Hospital, Taipei Medical University, New Taipei City, Taiwan; 2 Department of Neurology, School of Medicine, College of Medicine, Taipei Medical University, Taipei, Taiwan; 3 Taipei Neuroscience Institute, Taipei Medical University, Taipei, Taiwan; 4 College of Medical Science and Technology, Graduate Institute of Neural Regenerative Medicine, Taipei Medical University, Taipei, Taiwan

**Keywords:** Parkinson’s disease, PD, extracellular vesicle, EV, biomarkers, neurofilament light chain, NfL

## Abstract

Parkinson’s disease (PD) is a common neurodegenerative disorder characterized by progressive symptoms, underscoring the urgent need for predictive blood biomarkers. Plasma extracellular vesicles (EVs) offer a promising platform for biomarker development, with neurofilament light chain (NfL) emerging as a potential candidate for neurological diseases. This study evaluated plasma EV NfL as a biomarker for disease progression in a PD cohort. A total of 55 patients with PD (PwP) and 58 healthy controls (HCs) were followed, with PwP completing an average of 3.96 visits and HCs 2.25 visits. Plasma EVs were isolated and validated, and EV NfL levels were measured using an immunomagnetic reduction assay. Generalized estimating equations and Spearman correlations assessed relationships between clinical symptom progression and biomarkers. Although no significant differences in plasma EV NfL levels were observed between PwP and HCs over time, changes in plasma EV NfL significantly correlated with motor symptom progression, specifically with adjusted-total and akinetic-rigidity subscores of the Unified PD Rating Scale (UPDRS) Part III. Additionally, changes in UPDRS Part II scores were significantly associated with plasma EV NfL levels. These findings suggest that plasma EV NfL reflects motor symptom progression in PwP, highlighting its potential as a valuable biomarker for monitoring disease progression and guiding clinical trials in PD.

## Introduction

Parkinson’s disease (PD) is a chronic, progressive neurodegenerative disorder characterized by both motor symptoms and a range of non-motor symptoms, including dementia, depression, dysautonomia, and pain [1]. Despite significant advances in understanding the pathophysiology of PD, effective disease management and monitoring remain challenging [2]. A critical unmet need in PD research and clinical practice is the identification of reliable biomarkers that can accurately reflect disease progression [3]. Biomarkers are essential for early diagnosis, monitoring therapeutic efficacy, and predicting disease trajectories, ultimately enabling more personalized treatment strategies. Over the years, several mainstream targets, such as α-synuclein [4] and inflammatory markers [5], have been explored as potential biomarkers for PD. However, these efforts have often yielded inconsistent results due to variability in sensitivity, specificity, and reproducibility across different studies and populations. This inconsistency highlights the need for continued exploration and validation of novel biomarkers that can address these limitations. Neurofilament light chain (NfL) is a cytoskeletal protein predominantly found in neurons. Physiologically, NfL provides structural support and helps maintain axonal caliber, which is crucial for the proper conduction of nerve impulses. Pathologically, elevated levels of NfL in bodily fluids, such as cerebrospinal fluid (CSF) and blood, indicate neuronal damage and axonal degeneration [6]. As neurons degenerate, NfL is released into the extracellular space and eventually enters the bloodstream, making it a promising biomarker for various neurological disorders [7].

Previous studies have extensively examined the utility of NfL as a biomarker for various neurological diseases, including multiple sclerosis (MS) [8], amyotrophic lateral sclerosis (ALS) [9], and Alzheimer’s disease (AD) [10]. These studies have demonstrated that elevated NfL levels correlate with disease severity, progression, and response to therapy, underscoring its potential as a reliable biomarker. For example, in MS, higher NfL levels are associated with increased disease activity and worse clinical outcomes. Similarly, in ALS, elevated NfL levels have been linked to faster disease progression and shorter survival. In AD, higher NfL levels are observed in patients with more severe cognitive impairment and greater neurodegeneration. In the context of PD, recent research has also explored NfL as a potential biomarker. Studies indicate that blood NfL levels are higher in PD patients compared to healthy controls (HCs), with some evidence suggesting these levels may correlate with disease severity and progression [11–13]. While these findings are promising, the results remain somewhat variable, necessitating further research to validate NfL as a robust biomarker for PD progression. One of the major challenges in identifying reliable biomarkers for neurological diseases in peripheral blood is the need for markers that accurately reflect central nervous system (CNS) pathology while remaining accessible. In early PD, the intact BBB presents a particular challenge, as it restricts the movement of CNS-derived molecules into the bloodstream [14]. This results in low concentrations of CNS-specific biomarkers in peripheral blood, complicating their detection and diagnostic utility. Blood extracellular vesicles (EVs), particularly exosomes, have emerged as a promising source of biomarkers due to their ability to cross the BBB [15] and their remarkable stability in the bloodstream [16]. EVs are small, membrane-bound particles released by cells that carry biomolecules, such as proteins, lipids, and RNAs, providing valuable insights into the state of their cells of origin [17]. The development of blood EV biomarkers has shown significant potential in neurological diseases due to several key advantages: they can be collected non-invasively, their cargo is protected from enzymatic degradation, and they can reflect dynamic changes in the CNS [18, 19]. Additionally, EVs serve as cell-specific messengers, often carrying unique molecular signatures that reflect specific cellular processes or pathologies. This specificity makes them particularly useful for identifying subtle pathophysiological changes in diseases, such as PD and AD, where direct access to CNS tissue is highly challenging [20]. Recent advancements in EV isolation and characterization techniques have further enhanced their utility, enabling the identification of disease-associated proteins and nucleic acids with high sensitivity and specificity [21]. These developments position EVs as a promising avenue for biomarker discovery in neurological diseases. With the aforementioned advantages, blood EV NfL has been studied as a biomarker in several neurological diseases. Research indicates that exosomal NfL levels are elevated in veterans with a history of mild traumatic brain injury compared to controls [22, 23]. In genetic forms of frontotemporal dementia, studies have shown that exosomal NfL levels correlate with disease severity [24]. These findings highlight the potential utility of EV NfL as a biomarker for diagnosing and tracking the progression of neurodegenerative diseases. In PD, a recent study investigated the potential of blood EV NfL as a biomarker. Findings from these studies demonstrated that NfL levels within blood EVs are similar in people with PD (PwP) compared to HCs. However, blood EV NfL levels were found to correlate with disease severity. Specifically, blood EV NfL in PwP showed a trend toward correlation with the severity of akinetic rigidity (AR), while PwP in the lowest quartile of EV NfL levels had lower Unified PD Rating Scale (UPDRS) Part III scores after adjustments for age, sex, and disease duration [25]. These findings suggest that blood EV NfL could serve as a reliable indicator of PD severity. While these results are promising, further research is needed to establish standardized protocols for EV isolation and NfL measurement, as well as to validate these findings in larger, longitudinal cohorts. In the present study, we focus on a single-center cohort of PwP, with annual follow-up assessments of both motor and cognitive functions in individuals with PD and control subjects. The primary objective of this study is to investigate blood EV biomarkers, with a particular emphasis on NfL. By analyzing blood EV NfL levels, we aim to delineate their association with the clinical progression of PD. This study seeks to provide valuable insights into the potential of blood EV NfL as a reliable biomarker for monitoring disease progression, ultimately contributing to improved management and treatment strategies for individuals living with PD.

## Materials and methods

### Study population

Since November 2017, the study has been recruiting participants, including people with PwP and HCs, from Taipei Medical University-Shuang Ho Hospital. The initial cohort was established with the approval of the Joint Institutional Review Board of Taipei Medical University (approval nos. N201609017 and N201801043). PD is diagnosed using the UK Parkinson’s Disease Society Brain Bank Diagnostic Criteria. Enrollment of PwP is limited to those in the early to mid-stages of the disease, defined by Hoehn and Yahr stages I–III. HCs must be free from significant neurodegenerative diseases and disabilities and are regularly monitored in outpatient clinics for chronic conditions, such as hypertension, diabetes, hyperlipidemia, headaches, or vertigo. To date, 140 PwP and 66 HCs have completed their initial visits. The dropout rate was notably high during the COVID-19 pandemic. Since 2022, a new cohort, approved by the Joint Institutional Review Board of Taipei Medical University (approval no. N202205008), has been responsible for ongoing recruitment and follow-up of pre-enrolled PwP and HCs. This cohort has previously examined the cross-sectional results of plasma EV NfL in PD [25]. The current study now emphasizes longer-term follow-up, including only PwP with three or more visits and HCs with two to three visits in the analysis.

### Clinical assessments

Each participant undergoes an interview to collect baseline demographic information. Cognitive function is assessed using the Taiwanese versions of the Mini-Mental State Examination (MMSE) and the Montreal Cognitive Assessment (MoCA), both administered by trained nurses. The MMSE is a widely used screening tool for evaluating global cognitive function. It assesses various cognitive domains, making it useful for identifying cognitive impairments and monitoring changes over time. The exam is quick and easy to administer (∼10 min) but is less sensitive in detecting mild cognitive impairment (MCI) and is influenced by the participant’s level of education [26]. In contrast, the MoCA is a more sensitive tool for identifying MCI and early cognitive changes. It provides a more comprehensive evaluation than the MMSE, particularly in assessing executive functions and attention. However, it takes slightly longer to administer (∼10–15 min) and requires proper training to ensure accurate administration [27]. Additionally, all participants are evaluated during an outpatient visit using Parts I, II, and III of the UPDRS. The UPDRS is a comprehensive tool designed to assess and monitor the severity of both motor and non-motor symptoms in PD. It allows for a standardized measure to track disease progression and response to therapy. Of the UPDRS sections, Part II (Activities of Daily Living) and Part III (Motor Examination) are particularly important for evaluating functional and motor symptoms. Part II assesses how PD symptoms impact a patient’s daily life and ability to perform routine tasks. Patients rate their difficulties over the previous week, with caregivers sometimes providing input. Part III focuses on motor functions and involves a physical examination conducted by a healthcare professional. This section provides an objective clinical evaluation of motor symptoms, offering valuable insights into the severity and progression of the disease [28]. The interval between the last dose of anti-PD medication and the UPDRS Part III assessment is not documented, so it is assumed that patients with PD are assessed in their “on” state. Subscores for tremor, AR, and postural instability and gait disturbance (PIGD) are derived from the UPDRS Part III subitems and calculated based on modifications from a previous study [29].

### Isolation, concentration, and validation of EVs

Venous blood samples are collected and centrifuged at 13,000 × *g* for 20 min to obtain serum. Exosome isolation is performed on 1 mL of serum using the exoEasy Maxi Kit, following the manufacturer’s instructions. The final elution step yields 400 µL of eluate, which is concentrated to 100 µL using a Nanosep centrifugal device equipped with a 10K Omega membrane for subsequent analysis. The characterization of the isolated EVs as exosomes has been previously described [30], including detection of tetraspanins (CD9, CD63, CD81), TSG 101, and the absence of mitochondrial protein and cytochrome c, along with size distribution measured via nanoparticle tracking. For Western blot analysis, use specific antibodies against CD9, CD63, CD81, TSG 101, cytochrome c, and HSP70 at a 1:1000 dilution. Nanoparticle tracking analysis is conducted using a NanoSight NS300, following the manufacturer’s guidelines.

### Immunomagnetic reduction (IMR) assay for quantification of NfL

The details of the IMR assay used to quantify plasma EV NfL have been previously described [25] and were performed by MagQu Co. As specified in the instructions provided by MagQu Co., the assay’s detection limit ranges from 0.0033 to 1000 pg/mL.

### Ethical statement

The study with the approval of the Joint Institutional Review Board of Taipei Medical University (approval no. N201609017, N201801043 and N202205008).

### Statistical analysis

Conduct statistical analyses using IBM SPSS (version 26; IBM, Armonk, NY, USA). Utilize generalized estimating equations (GEEs) to evaluate the associations between plasma EV NfL levels and the progression of clinical symptoms. Assess the relationship between plasma EV NfL and age- and sex-adjusted clinical symptoms in PwP using Spearman correlation. To account for variations in age and gender across different visits, standardize the UPDRS, MMSE, and MoCA scores into Z scores. For this, calculate the mean and standard deviation of these scores within each age and gender group. Then, convert individual scores into Z scores to indicate how many standard deviations a score is from the group mean. Positive Z scores represent scores above the group mean, while negative Z scores represent scores below the group mean. Finally, consider *P* values less than 0.05 as statistically significant throughout the analyses.

## Results

### Demographic information

**Figure 1.**
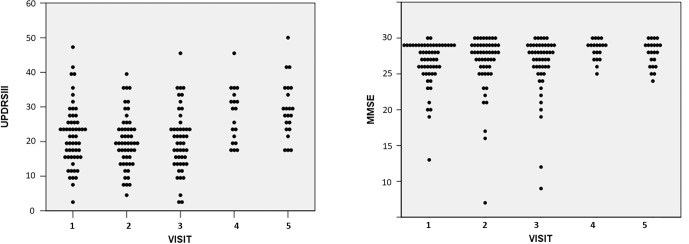
(A) The progression of motor symptoms (assessed by total score of UPDRS part III) and (B) cognition (assessed by MMSE) in people with Parkinson’s disease. Data was presented as dot plot. MMSE: Mini-Mental State Examination; UPDRS: Unified Parkinson’s Disease Rating Scale.

Clinical and plasma EV biomarker data were analyzed from 55 PwP with three or more visits and 58 HCs with two or more visits. No significant differences were observed in age, gender distribution, baseline MMSE, or MoCA scores between PwP and HCs (Table 1). In terms of disease progression, UPDRS III scores for PwP remained stable during the first and second follow-ups compared to baseline but showed a substantial decline at the third follow-up. For cognitive performance, MMSE scores in PwP did not exhibit significant changes throughout the follow-up period (Figure 1A and 1B).

**Table 1:** Baseline demographic data of study participants (with completed baseline and follow-up)

	**HCs, *n* ═ 58**	**PwP, *n* ═ 55**	***P* value (HCs vs PwP)**
Age	67 (4.25)	69 (4)	0.13
Male:Female	36:22	30:25	0.45
Baseline			
MMSE	28 (1.5)	27 (2)	0.11
MoCA	24 (3.125)	23 (3)	0.23
UPDRS-II		6 (3.5)	–
UPDRS-III		22 (5.5)	–

Data was presented as median (interquartile range). HC: Healthy control; PwP: People with Parkinson’s disease; MMSE: Mini-Mental State Examination; MoCA: Montreal Cognitive Assessment; UPDRS: Unified Parkinson’s Disease Rating Scale.

### The change of plasma EV NfL in PwP and HCs

**Figure 2.**
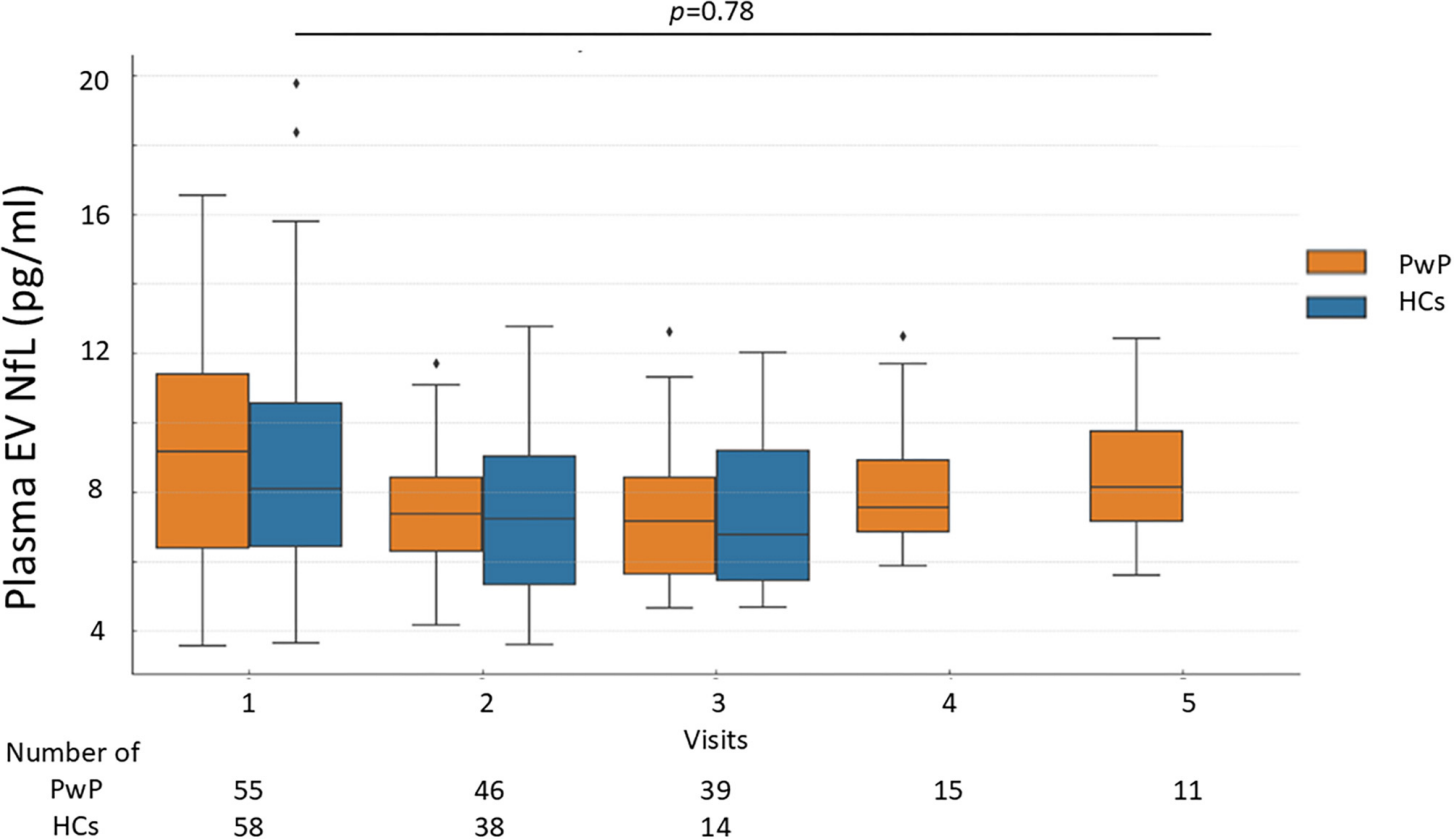
**Levels of plasma EV NfL in PwP and HCs.** Generalized estimating equations were utilized to compare the change of plasma EV NfL between PwP with HCs. Data are presented as median (interquartile range). EV: Extracellular vesicle; NfL: Neurofilament light chain; HC: Healthy control; PwP: People with Parkinson’s disease.

At baseline, there was no significant difference in plasma EV NfL levels between PwP and HCs. Similarly, during follow-up, the change in plasma EV NfL levels did not differ significantly between PwP and HCs after adjusting for age and sex (Figure 2).

### Clinical motor and cognition

Taking only PwP into account, the overall trend in the change of plasma EV NfL was positively associated with changes in UPDRS II during the follow-up period. However, no association was found between the overall change in plasma EV NfL and UPDRS III (total or subscore), MMSE, or MoCA (Table 2).

### Correlation of changes in plasma EV NfL with changes in clinical motor and cognition severity

**Figure 3.**
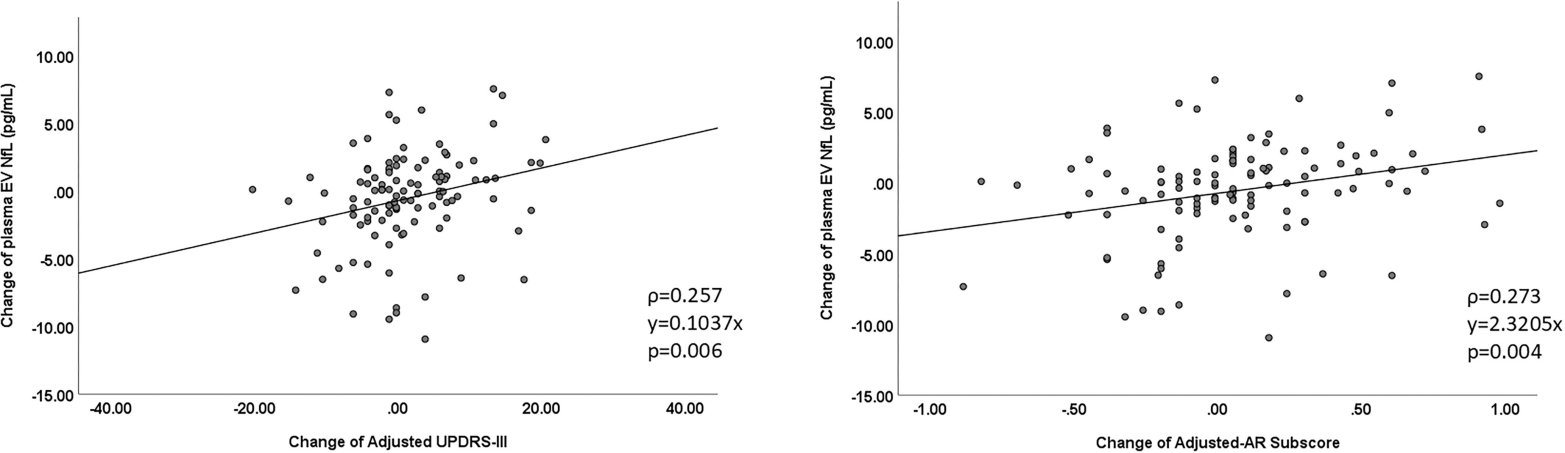
The association between the change of plasma extracellular vesicle neurofilament light chain with the change of adjusted UPDRS part III (A), and the change of adjusted MMSE (B). The UPDRS part III and MMSE were standardized to Z scores with the adjustment of age and gender variations across different visits. EV: Extracellular vesicle; NfL: Neurofilament light chain; MMSE: Mini-Mental State Examination; UPDRS: Unified Parkinson’s Disease Rating Scale; AR: Akinetic rigidity.

To comprehensively examine the relationship between changes in plasma EV NfL levels and clinical progression, a correlation analysis was conducted at each visit time point. A significant association was observed between changes in plasma NfL levels and changes in the age- and sex-adjusted total UPDRS III scores (ρ ═ 0.257, *P* ═ 0.006), as well as AR subscores (ρ ═ 0.273, *P* ═ 0.004) at each follow-up time point (Figure 3A and 3B). However, plasma EV NfL levels at each visit were not associated with clinical progression at the subsequent visit in PwP (data not shown).

**Table 2:** Association between the changes in plasma EV NfL level with the changes in clinical assessments in people with Parkinson’s disease after adjustment of age and sex

	**Changes in plasma EV NfL**		
	**β**	**95% CI**	***P* value**
**Changes in**			
UPDRS-II	−0.30	−0.55 ∼ -0.05	0.017
UPDRS-III	0.28	−0.16 ∼ 0.73	0.212
MMSE	−0.004	−0.15 ∼ 0.14	0.954
MoCA	−0.04	−0.17 ∼ 0.09	0.559

EV: Extracellular vesicle; NfL: Neurofilament light chain; CI: Confidence interval; MMSE: Mini-Mental State Examination; MoCA: Montreal Cognitive Assessment; UPDRS: Unified Parkinson’s Disease Rating Scale.

## Discussion

In this study, we investigated a single-center cohort of PwP. During the follow-up period, blood EV NfL levels fluctuated in both PwP and HCa, with no significant differences in the patterns of change between the two groups. Notably, within the PD cohort, changes in UPDRS II scores were positively and significantly associated with changes in blood EV NfL levels. Furthermore, fluctuations in blood EV NfL levels across different time points were significantly correlated with age- and sex-adjusted changes in UPDRS III scores, as well as the AR subscore within UPDRS III. These findings highlight the potential of blood EV NfL as a biomarker for tracking the progression of PD motor symptoms. This study provides valuable insights into the relationship between blood EV NfL and the clinical progression of PD, contributing to the advancement of management and treatment strategies for individuals with PD.

NfL has been extensively studied as a biomarker for PD across various research efforts [13], including significant findings from the Parkinson’s Progression Markers Initiative (PPMI) study. This large-scale, longitudinal cohort study has consistently demonstrated that NfL levels are elevated in PD patients compared to HCs [11, 31]. Moreover, higher NfL levels in PD patients have been linked to faster disease progression, greater motor impairment, and cognitive decline [32–35]. These findings suggest that NfL could serve as a reliable indicator of disease severity and progression in PD. The rationale for changes in NfL levels as PD progresses lies in the underlying pathophysiological processes. NfL, a structural component of neurons—particularly within axons—is released into the extracellular space and subsequently into the bloodstream in response to neuronal damage and axonal degeneration [36]. As PD advances, the growing neurodegenerative burden likely leads to increased axonal damage, resulting in elevated NfL levels. This relationship between NfL levels and disease progression underscores its potential utility in monitoring PD.

The role of NfL within EVs has garnered significant attention in recent literature [24, 37, 38]. EVs carry various molecular cargo, including proteins like NfL. The encapsulation of NfL within EVs provides a protected environment that facilitates its transport across the BBB, making it detectable in peripheral blood. This property enhances the potential of EV-contained NfL as a biomarker for neurological diseases, including PD. Studies have investigated whether the presence of NfL in EVs reflects axonal loss or regeneration. The prevailing evidence indicates that elevated levels of EV-contained NfL primarily signal axonal damage and degeneration [22]. This aligns with broader findings showing increased NfL levels in various neurodegenerative conditions, where NfL is associated with disease severity and progression. In the context of PD, the correlation between higher EV-contained NfL levels and worsening motor symptoms further supports the notion that these elevated levels serve as markers of ongoing neurodegeneration rather than regeneration. If EV-contained NfL were reflective of axonal regeneration, its levels would be expected to correlate with clinical symptom improvement. However, the observed positive association between EV-contained NfL levels and the deterioration of motor symptoms suggests that its presence is more likely a consequence of neurodegenerative processes. The release of NfL into EVs may result from neuronal injury, where damaged axons shed NfL into the extracellular space, followed by its encapsulation in EVs via active secretion mechanisms [39]. This process likely represents a cellular strategy for managing and disposing of damaged components rather than a sign of axonal repair. Overall, the presence of NfL in EVs mirrors the extent of neuronal damage and axonal degeneration in PD. As such, EV-contained NfL is a valuable biomarker for tracking disease progression and motor symptom deterioration, offering insights into the underlying neurodegenerative processes driving PD. The urgent need for reliable progression biomarkers in PD is underscored by challenges in monitoring clinical trial outcomes. Current assessments of motor symptoms, such as the UPDRS, can be influenced by physical conditions unrelated to PD, potentially confounding the interpretation of trial results. In contrast, blood EV-contained NfL levels provide a more stable and specific biomarker, less susceptible to variations caused by systemic conditions. This stability makes blood EV-contained NfL an invaluable tool for accurately monitoring disease progression and evaluating therapeutic interventions. Motor symptom scales like the UPDRS often struggle to detect subtle changes during treatment, limiting their sensitivity in assessing the efficacy of potential therapies. Blood EV-contained NfL, however, can capture these nuanced changes, providing a more precise measure of disease dynamics. This capability is particularly critical in early-phase clinical trials, where identifying small but significant alterations can inform the therapeutic potential of investigational treatments. Among PD motor symptoms, AR is strongly associated with Lewy body pathology in the brains of PD patients [40]. The observed correlation between changes in adjusted AR subscores and blood EV-contained NfL levels suggests that this biomarker reflects underlying disease pathology. Additionally, the present study demonstrated a significant association between changes in the UPDRS Part II score and plasma EV-contained NfL levels. Unlike the UPDRS Part III, which is influenced by medication, the UPDRS Part II provides a more reliable assessment of motor symptom progression in PD [41]. It reflects the severity of PD and is strongly linked to its pathology, particularly the loss of dopaminergic neurons. These findings underscore the potential of blood EV-contained NfL as a key biomarker in understanding neuronal loss underlying PD pathology. In conjunction with other blood EV biomarkers, such as α-synuclein, which target disease-specific mechanisms, NfL could greatly enhance our ability to track disease progression and therapeutic outcomes.

This relationship further supports the potential utility of blood EV NfL in clinical trials aimed at disease modification. By providing a sensitive and specific measure of neurodegeneration, blood EV NfL could play a pivotal role in evaluating the effectiveness of therapies designed to alter the course of PD. In summary, the integration of blood EV NfL as a biomarker in clinical trials offers a promising approach to enhancing the precision of disease monitoring and therapeutic assessment, ultimately advancing the development of effective treatments for PD. The study had several notable limitations. First, the use of a single-center cohort limits the generalizability of the findings, as the results may not apply to populations outside the study’s demographic and environmental context. Second, the study’s longitudinal data is limited, with participants having varying numbers of follow-up visits. This inconsistency may impact the robustness of conclusions regarding the progression of plasma EV NfL levels and their association with PD symptoms. Third, a significant dropout rate during the COVID-19 pandemic may have introduced bias, affecting the consistency and reliability of the data, and potentially influencing the study’s overall findings. Fourth, there were concerns that the EV NfL detected might not solely originate from neuronal damage but could also stem from aggregates or external associations with EVs. The present study utilized commercially available size exclusion chromatography for EV isolation, which was not entirely free from contaminant co-isolation. Furthermore, NfL, being a cytoskeletal protein, can form aggregates under certain conditions, potentially complicating its interpretation as a biomarker for neuronal injury. Lastly, the absence of an observed association between blood EV NfL and other common PD biomarkers, particularly α-synuclein, along with the limited availability of animal studies to confirm the correlation between blood EV NfL and PD pathology, weakens the evidence supporting the relevance of blood EV NfL to the underlying pathology of PD. However, the study provided valuable insights into the relationship between changes in plasma EV NfL levels and specific motor symptoms in PD, such as those measured by the UPDRS Part III. These findings underscore the potential of plasma EV NfL as a reliable indicator of motor progression in PD, which could inform future clinical trials and therapeutic strategies aimed at modifying disease progression. In addition, despite its suboptimal association with PD severity, blood EV NfL may complement other EV biomarkers of PD, forming a panel that encompasses both disease mechanisms and pathology to enhance prognosis prediction.

## Conclusion

In conclusion, this study underscores the potential of plasma EV NfL as a promising biomarker for monitoring PD progression. By revealing significant associations between changes in EV NfL levels and motor symptom progression, the research offers valuable insights into the underlying neurodegenerative processes of PD. These findings suggest that EV NfL could serve as a non-invasive, reliable biomarker for tracking disease progression. Moreover, the results provide a solid foundation for future studies to validate the clinical utility of EV NfL in PD, with the potential to enhance disease management and treatment strategies.

## Data Availability

The availability of data and materials required permission from the TMU-JIRB.
